# Supplementary material for: Designing online species identification tools for biological recording: the impact on data quality and citizen science learning
Source: PeerJ. 2019 Jan 28;6:e5965. doi: 10.7717/peerj.5965 (PMC6354666; doi:10.7717/peerj.5965)
Supplement: Supplemental Information 2 [file peerj-07-5965-s002.docx]

# Appendices

### Appendix 1: Experimental design

Table 1a: **P** represents images (numbered 1-6 where 1-3 are easy and 4-6 difficult ) and **I** represents Interface (numbered 1-3 where 1=Field guide 2=Feature selection and 3= Decision Tree). The table shows the order of execution of tasks by each participant to counterbalance the order effects so that each task occupied each position in the order of task execution at least once.

| Participant No | Task 1 | Task 2 | Task 3 | Task 4 | Task 5 | Task 6 |
| --- | --- | --- | --- | --- | --- | --- |
| 1 | P1 I1 | P2 I2 | P3 I3 | P4 I1 | P5 I2 | P6 I3 |
| 2 | P2 I2 | P3 I3 | P4 I1 | P5 I2 | P6 I3 | P1 I1 |
| 3 | P3 I3 | P4 I1 | P5 I2 | P6 I3 | P1 I1 | P2 I2 |
| 4 | P4 I1 | P5 I2 | P6 I3 | P1 I1 | P2 I2 | P3 I3 |
| 5 | P5 I2 | P6 I3 | P1 I1 | P2 I2 | P3 I3 | P4 I1 |
| 6 | P6 I3 | P1 I1 | P2 I2 | P3 I3 | P4 I1 | P5 I2 |
| 7 | P1 I2 | P2 I3 | P3 I1 | P4 I2 | P5 I3 | P6 I1 |
| 8 | P2 I3 | P3 I1 | P4 I2 | P5 I3 | P6 I1 | P1 I2 |
| 9 | P3 I1 | P4 I2 | P5 I3 | P6 I1 | P1 I2 | P2 I3 |
| 10 | P4 I2 | P5 I3 | P6 I1 | P1 I2 | P2 I3 | P3 I1 |
| 11 | P5 I3 | P6 I1 | P1 I2 | P2 I3 | P3 I1 | P4 I2 |
| 12 | P6 I1 | P1 I2 | P2 I3 | P3 I1 | P4 I2 | P5 I3 |
| 13 | P1 I3 | P2 I1 | P3 I2 | P4 I3 | P5 I1 | P6 I2 |
| 14 | P2 I1 | P3 I2 | P4 I3 | P5 I1 | P6 I2 | P1 I3 |
| 15 | P3 I2 | P4 I3 | P5 I1 | P6 I2 | P1 I3 | P2 I1 |
| 16 | P4 I3 | P5 I1 | P6 I2 | P1 I3 | P2 I1 | P3 I2 |
| 17 | P5 I1 | P6 I2 | P1 I3 | P2 I1 | P3 I2 | P4 I3 |
| 18 | P6 I2 | P1 I3 | P2 I1 | P3 I2 | P4 I3 | P5 I1 |

### Appendix 2: NASA TLX workload questionnaire

***NASA Task Load Index***

*Hart and Staveland’s NASA Task Load Index (TLX) method assesses work load on five 7-point scales. Increments of high, medium and low estimates for each point result in 21 gradations on the scales.*

Mental Demand How mentally demanding was the task?


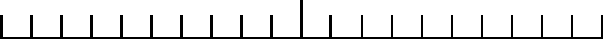


Very Low Very High

Physical Demand How physically demanding was the task?


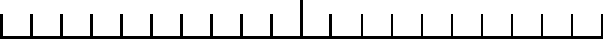


Very Low Very High

Temporal Demand How hurried or rushed was the pace of the task?


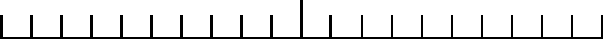


Very Low Very High

Performance How successful were you in accomplishing what

you were asked to do?


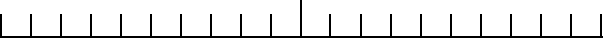


Perfect Failure

Effort How hard did you have to work to accomplish

your level of performance?


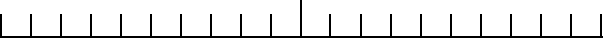


Very Low Very High

Frustration How insecure, discouraged, irritated, stressed,

and annoyed were you?


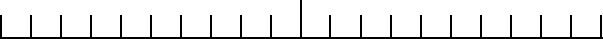


Very Low Very High

| Effort  or  Performance | Physical Demand or Temporal Demand | Performance or  Temporal Demand |
| --- | --- | --- |
| Temporal Demand or  Frustration | Physical Demand  or  Performance | Mental Demand or  Effort |
| Temporal Demand or Effort | Temporal Demand  or  Mental Demand | Mental Demand or  Physical Demand |
| Physical Demand or  Frustration | Frustration or  Effort | Effort  or  Physical Demand |
| Performance  or  Frustration | Performance or  Mental Demand | Frustration or  Mental Demand |
